# Application of DNA barcoding confirms the host of *Gonatopus viet* Olmi, 1986 (Hymenoptera, Dryinidae)

**DOI:** 10.3897/zookeys.944.53054

**Published:** 2020-06-30

**Authors:** Hua-Yan Chen, Massimo Olmi, Hong Pang, Jing-Xian Liu

**Affiliations:** 1 State Key Laboratory of Biocontrol, School of Life Sciences / School of Ecology, Sun Yat-sen University, Guangzhou 510275, China Sun Yat-sen University Guangzhou China; 2 Tropical Entomology Research Center, Via De Gasperi 10, I-01100, Viterbo, Italy Tropical Entomology Research Center Viterbo Italy; 3 Department of Entomology, South China Agricultural University, Guangzhou 510642, China South China Agricultural University Guangzhou China

**Keywords:** Chrysidoidea, host association, leafhopper, molecular identification, *Stirellus capitatus*

## Abstract

*Gonatopus viet* Olmi, 1986 was originally described from Vietnam based on a single female. No further distribution records or hosts have been documented since its original discovery. In the present study, this species is newly recorded from China and its host is confirmed as *Stirellus capitatus* (Distant, 1918) using DNA barcoding techniques. The utility of DNA barcoding to discover host-Dryinidae associations is discussed.

## Introduction

Species of Dryinidae (Hymenoptera: Chrysidoidea) are parasitoids and predators of Auchenorrhyncha (Hemiptera), many of which are important insect pests in agriculture and forestry (Olmi 1994). These wasps are considered to be important biological control agents against Auchenorrhyncha pests and a number of them have been deployed for that purpose (Guglielmino and Olmi 1997; He and Xu 2002; Guglielmino et al. 2013; Vétek et al. 2019). Further development of their potential use as biological control agents depends upon a better understanding of the biology and hosts of these wasps (Guglielmino et al. 2013). However, our knowledge on host-Dryinidae interactions usually requires the rearing of the wasps from their hosts, which is difficult, if not impossible, for many dryinid species due to the rarity of the wasps themselves or difficulties in keeping the parasitized host alive until the emergence of the wasps.

On the other hand, most species of Dryinidae are ectoparasitoids of the nymphs or adults of their hosts, that is, the larvae of the wasps strongly protrude from the host’s body and feed on the internal structures of the host from outside (Olmi 1994). These parasitized hosts as well as the adults of Dryinidae wasps and hosts are often captured by Malaise and yellow pan traps, rendering a rich resource for exploring the host associations of Dryinidae. While it is difficult to identify the host in the nymphal stage and the wasp species based on its larval morphology, DNA barcoding techniques may have a great potential to close this gap. The barcode region of the mitochondrial cytochrome oxidase subunit 1 (COI) could be used to identify all life states of animals (Hebert et al. 2003).

**Figure 1.**
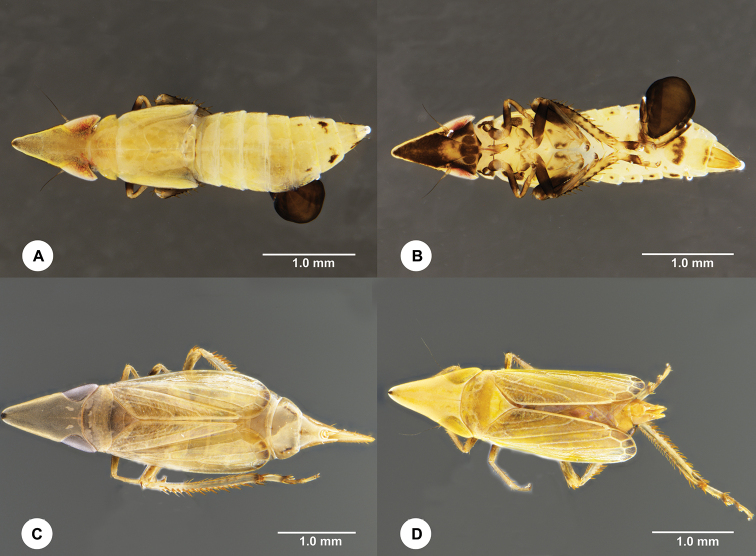
**A, B***Stirellus capitatus* (Distant, 1918) nymph parasitized by *Gonatopus viet* Olmi, 1986 (SCAU 3040955) **A** habitus, dorsal view **B** habitus, ventral view **C, D***Stirellus capitatus* (Distant, 1918) **C** habitus, female (SCAU 3040956), dorsal view **D** habitus, male (SCAU 3049598), dorsal view.

During an expedition to the Xisha Islands of South China Sea organized by Sun Yat-sen University in 2019, the first author collected a *Gonatopus* adult, a parasitized leafhopper nymph (Figure 1A, B) and many adults of the leafhopper species in yellow pan traps on the small Dong Island. In this study, we use DNA barcoding to confirm that the leafhopper, *Stirellus capitatus* (Distant, 1918) (Hemiptera: Cicadellidae), is the host of *Gonatopus viet* Olmi, 1986, on Dong Island.

## Materials and methods

This work is based upon specimens of Dryinidae wasps and leafhoppers collected by yellow pan traps (YPT) on Dong Island (16°39.875'N, 112°43.813'E) of South China Sea. The adult dryinid wasp was identified using the keys of Xu et al. (2013). The adult leafhoppers were identified using the descriptions of Duan et al. (2016). The Dryinidae larva was extracted from the host body and analyzed. All the studied specimens are deposited in the Museum of Biology at Sun Yat-sen University, Guangzhou, China (**SYSBM**). Images and measurements were made using Nikon SMZ25 microscope with a Nikon DS-Ri 2 digital camera system. Images were post-processed with Adobe Photoshop CS6 Extended.

Genomic DNA was extracted from the adult wasp, the wasp larva, the parasitized leafhopper nymph, and a female leafhopper adult. A nondestructive DNA extraction protocol was used for the adult wasp and leafhopper, as described in Taekul et al. (2014), to enable preservation of a voucher specimen. For the wasp larva and the leafhopper nymph, only the skin and a single leg were used, respectively. DNA was extracted using a DNeasy Blood & Tissue Kit (QIAGEN, Inc.) and LCO1490 and HCO2198 primers (Folmer et al. 1994) were used to amplify the COI sequences. Amplicons were sequenced on an Applied Biosystems (ABI) 3730XL by Sangon Biotech (Shanghai, China). Chromatograms were assembled with Geneious 11.0.3. All the amplified sequences were deposited into GenBank (Table 1). Sequences were aligned and compared in Geneious 11.0.3 using the MAFFT alignment algorithm.

**Table 1.** Details of taxon sampling, codes, and accession numbers.

**Taxon**	**Code**	**Accession number**
*Gonatopus viet* female adult	SCAU 3040953	MT311154
Dryinidae larva	SCAU 3040954	MT311155
*Stirellus capitatus* nymph	SCAU 3040955	MT311156
*Stirellus capitatus* female adult	SCAU 3040956	MT311157

## Results

**Figure 2.**
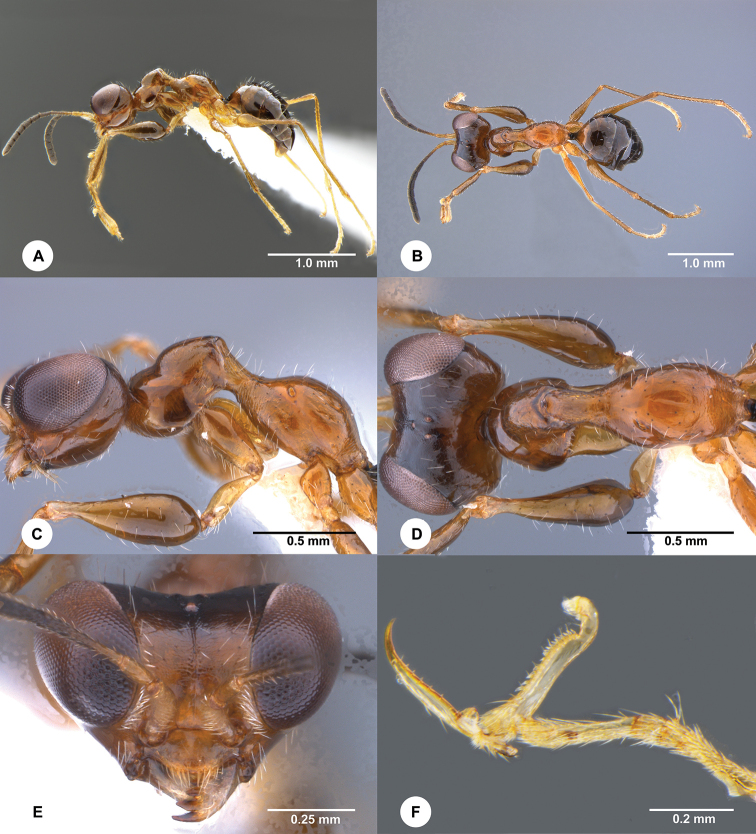
*Gonatopus viet* Olmi, female (SCAU 3040953) **A** habitus, lateral view **B** habitus, dorsal view **C** head and mesosoma, lateral view **D** head and mesosoma, dorsal view **E** head, anterior view **F** chela.

The Dryinidae female adult is identified as *G. viet* based on morphology. It matches well with the original descriptions of *G. viet* except the body length is 2.86 mm (the holotype is 2.4 mm). This species can be recognized by the following characters (Figure 2): apterous; head excavated, unsculptured; frontal line complete; palpal formula 4:2; pronotum shiny, setose, unsculptured, crossed by strong transverse impression; metanotum not hollow behind mesoscutellum; metathorax and metapectal-propodeal complex shiny, completely smooth, with propodeal declivity transversely striate only near distal apex; mesopleuron and metapleuron not transversely striate; enlarged claw with one small subapical tooth and seven peg-like hairs; protarsomere 5 with inner margin proximally not serrated, with one row of 15 lamellae; distal apex with about ten lamellae; tibial spurs 1/0/1. The COI sequences are over 99% identical between the adult wasp and the larva feeding on the leafhopper nymph, indicating that the wasp larva is conspecific with the adult wasp, i.e., *G. viet*.

The COI sequences are also over 99% identical between the parasitized leafhopper nymph and a female leafhopper adult, indicating that they are conspecific. This female leafhopper adult, along with the males from the same sample, is morphologically identified as *Stirellus capitatus* (Figure 1C, D), which has been previously recorded from South China, including Hainan Island.

## Discussion

Dryinidae is a diverse group of parasitic wasps, with approximately 1900 species described worldwide (Xu et al. 2013; Olmi and Xu 2015; Olmi et al. 2019). Guglielmino et al. (2013) compiled the most recent host-parasite catalogue of the world Dryinidae and recognized 1014 relationships between dryinids and their hosts. However, considering the species diversity of Dryinidae, this number is far from the actual interactions between these wasps and their hosts. Besides, dryinids are not monophagous; in contrast, they may parasitize different groups of hosts. The many current monophagous parasitism records may due to the insufficient investigation of host-parasitoid interactions. In this study, we successfully use DNA barcoding to identify the host of a dryinid wasp species, *G. viet*, and to match the nymphal and adult stages of the host, *S. capitatus*. The present study illustrates the great potential that DNA barcoding has for accelerating the discovery of host-Dryinidae associations. It is worth noting that in some cases dryinid larvae protruding from the host’s bodies contain hyperparasitoid larvae (Vétek et al. 2019) and may result in extracting mixture DNA from the wasp larvae. In such cases, taxon-specific primers (Mita et al. 2013) should be used or the Next Generation Sequencing approach should be applied.

Many species of Dryinidae display extreme sexual dimorphism, especially in the subfamilies Dryininae and Gonatopodinae (Mita and Matsumoto 2012; Tribull 2015). DNA markers have been shown to be powerful tools for the correct association of the female and male of Dryinidae species with sexual dimorphism (Mita and Matsumoto 2012), exploring intraspecific genetic variation (Mita et al. 2013), and molecular phylogeny (Tribull 2015). As the DNA barcode library of Dryinidae is populated with both sexes of additional species, we should be able to accelerate the discovery of host-Dryinidae associations using the approach present in this study.
